# It takes a team: Generating evidence to define and foster collective competence in health professions education

**DOI:** 10.36834/cmej.69664

**Published:** 2020-09-23

**Authors:** Maryam Wagner, Tim Dubé, Carlos Gomez-Garibello

**Affiliations:** 1 McGill University, Quebec, Canada; 2 Université de Sherbrooke, Quebec, Canada

The focus of health professions education (HPE) rests on identifying and fostering the *individual* competencies of learners. Yet, in hospitals, healthcare is frequently managed by a variety of health professionals providing expertise across different domains. Patients rarely receive all their healthcare from a single health professional, however straightforward or uncomplicated their health concerns.^1^ Although individual competence is necessary to respond to healthcare needs, it is insufficient to provide the highest quality healthcare; notions reflected in a shift in recent literature that emphasizes the collective competence of teams.^2^ In this commentary, we place emphasis on the importance of collective competence and advancing the construct through empirical research, using multiple methods, by focusing on key characteristics and mechanisms of teamwork that will help to generate evidence for a robust definition to improve training programs in HPE, and ultimately improve the health of Canadians.

Collective competence refers to both the abilities of a team (sum of individual competencies), as well as the relationships and collaborative processes among its members (more than the sum of individual competencies), working together to deliver high quality healthcare. The Canadian Interprofessional Health Collaborative released the first pan-Canadian model of interprofessional competencies in 2010.^3^ However, there remains a dearth of empirical research in HPE to support and/or expand the definition of collective competence.

Drawing upon research on collective competence in other fields including sport psychology, human resource management, and military operations we identify key factors that may guide empirical research to advance our understanding of collective competence. Generating empirical evidence is essential for enhancing HPE programs, reducing inefficiencies in health systems and service delivery, and, most importantly, improving health outcomes for the millions of Canadians who require quality care.

We argue that researchers exploring collective competence should examine the characteristics of teamwork and its coordination with the mechanisms that facilitate the influx of information within teamwork that in turn are associated with effective healthcare. Teamwork has been defined as a set of cognitions, actions and motivations that each team member requires for the team to function effectively. These cognitions, actions and motivations facilitate coordinated actions, adaptive performance, and achievement of valued outcomes.^4^

The first of the teamwork mechanisms is *shared mental models*. Mental models refer to the shared and anticipated representations of team goals, individual responsibilities, and team coordination to reach a goal. Training teams of individuals together (team training) can contribute to building shared mental models of clinical tasks and situations, task environments, and interactions of team members and increasing team’s ability to function effectively in high stress situations. The second coordinating mechanism for effective team performance is *closed-loop communication*. It involves a cycle that starts with a sender emitting a message; followed by a receiver receiving the message, interpreting and acknowledge its reception; and finally, a follow-up executed by the sender to ensure the message was correctly received. The final coordinating mechanism is *mutual trust* which refers to a shared perception that individuals will perform particular actions relevant to the members of a team, and the recognition of the rights and interests of all the team members.

These three mechanisms help to articulate the presence of the “Big Five” components of teamwork contributing to team effectiveness: i) team leadership (ability to guide and structure experiences and facilitate coordination); ii) mutual performance monitoring (awareness of team functioning by monitoring others’ actions); iii) backup behaviour (allocation and re-allocation of resources when a difficulty is experience by a team member); iv) adaptability (ability to detect deviations from planned and expected action, and readjust actions accordingly); and v) team orientation (preference to work collectively and tendency to enhance individual performance by working with others).^5^ All of these components of teamwork and associated mechanisms need to be explored in healthcare.

Additionally, we argue the need to examine additional characteristics of teamwork related to role clarification, role valuing, affect, and co-regulation of learning to better understand the complexities of collective competence. Role clarification provides an understanding of how individuals define their roles and recognize their boundaries and practice standards within their discipline. Examining role valuing provides an opportunity to consider the contribution of the respectful understanding for others brings to the effectiveness of the team.

Affective variables may influence both teamwork and workplace performance and contribute to understanding the association between emotions, cooperativeness, task performance perception, problem solving, and development of trustful relationships. Finally, co-regulation is a complex, and highly relevant facet that demands examination in teamwork. Co-regulation involves self-awareness about individuals’ self-expectations and perspectives as well as those from others involved in a task. It may be explored on a continuum from “individual regulation within group” to “co-regulation as a group.” Communication among team members becomes paramount in co-regulation, as it constitutes the vehicle and the context in which interactions take place.

Due to the complexities of these characteristics and associated mechanisms, it is integral that a variety of research methods be employed in both simulations and workplace-based settings to contribute to the understanding of collective competence. For example, a combination of methods such as direct observation, self-reports, interviews and point-of-view recordings may be integrated to provide a multi-method, robust analysis. We strongly advocate for an integrative research paradigm that incorporates individual competence, team dynamics, regulative mechanisms, and affective aspects that only emerge in social interaction. This work necessitates incorporating a multiplicity of perspectives to explore and evaluate how teams function in clinical contexts. As such, we encourage researchers to contribute their perspectives and expertise, individually and collectively, to respond to this call to advance our understanding and appreciation of the role of collective competence in healthcare delivery.

## References

[1] Lingard L, McDougall A, Levstik M, Chandok N, Spafford MM, Schryer C Representing complexity well: a story about teamwork, with implications for how we teach collaboration. Medical education. 2012 9 46(9):869-77. 10.1111/j.1365-2923.2012.04339.x 22891907

[2] Lingard L, Sue-Chue-Lam C, Tait GR, Bates J, Shadd J, Schulz V, Heart Failure/Palliative Care Teamwork Research Group Pulling together and pulling apart: influences of convergence and divergence on distributed healthcare teams. Advances in Health Sciences Education. 2017 12 1;22(5):1085-99. 10.1007/s10459-016-9741-2 28116565 PMC5668127

[3] Canadian Interprofessional Health Collaborative. A national interprofessional competency framework. Vancouver:Canadian Medical Education Journal 2010 32 p. Available from: https://drive.google.com/file/d/1Des_mznc7Rr8stsEhHxl8XMjgiYWzRIn/view [Accessed DATE]

[4] Salas EE, Fiore SM Team cognition: Understanding the factors that drive process and performance. American Psychological Association; 2004 10.1037/10690-000

[5] Salas E, Sims DE, Burke CS Is there a “big five” in teamwork?. Small group research. 2005 10 36(5):555-99. 10.1177/1046496405277134

